# Indwelling time of peripherally inserted central catheters and incidence of bloodstream infections in haematology patients: a cohort study

**DOI:** 10.1186/s13756-022-01069-z

**Published:** 2022-02-17

**Authors:** M. G. Caris, N. A. de Jonge, H. J. Punt, D. M. Salet, V. M. T. de Jong, B. I. Lissenberg-Witte, S. Zweegman, C. M. J. E. Vandenbroucke-Grauls, M. A. van Agtmael, J. J. W. M. Janssen

**Affiliations:** 1 Department of Internal Medicine, Amsterdam UMC, Vrije Universiteit Amsterdam, De Boelelaan 1117, 1081 HV Amsterdam, The Netherlands; 2 Department of Medical Microbiology and Infection Control, Amsterdam UMC, Vrije Universiteit Amsterdam, Amsterdam, The Netherlands; 3 Department of Haematology, Amsterdam UMC, Vrije Universiteit Amsterdam, Amsterdam, The Netherlands; 4 Epidemiology and Data Science, Amsterdam UMC, Vrije Universiteit Amsterdam, Amsterdam, The Netherlands

**Keywords:** CLABSI, PICC, CVC, Indwelling time, Haematology

## Abstract

**Background:**

We aimed to assess whether longer indwelling time of peripherally inserted central catheters (PICC) increases risk of central line associated bloodstream infections (CLABSI) in haematology patients.

**Methods:**

Multicentre retrospective cohort study among haematology patients receiving PICCs between 2013 and 2015. Occurrence of CLABSI based on CDC definitions was assessed. We calculated incidence rates, determined risk factors for CLABSI and used Poisson regression models to assess the risk of developing CLABSI as a function of PICC dwell time. We compared diagnoses and treatment characteristics between 2013–2015 and 2015–2020.

**Results:**

455 PICCs placed in 370 patients were included, comprising 19,063 catheter days. Median indwelling time was 26 days (range 0–385) and CLABSI incidence was 4.0 per 1000 catheter days, with a median time to CLABSI of 33 days (range 18–158). Aplastic anaemia (AA) was associated with an increased risk of CLABSI; patients undergoing autologous stem cell transplantation (SCT) were less likely to develop CLABSI. In the unadjusted analysis, PICCs with an indwelling time of 15–28 days, 29–42 days, 43–56 days and > 56 days each had an increased CLABSI incidence rate ratio of 2.4 (1.2–4.8), 2.2 (0.95–5.0), 3.4 (1.6–7.5) and 1.7 (0.9–3.5), respectively, compared to PICCs in place for < 15 days. However, after adjusting for AA and SCT, there was no significant difference in incidence rates between dwell times (*p* 0.067).

**Conclusions:**

Our study shows that risk of CLABSI does not appear to increase with longer PICC indwelling time. Routine replacement of PICCs therefore is unlikely to prevent CLABSI in this population.

**Supplementary Information:**

The online version contains supplementary material available at 10.1186/s13756-022-01069-z.

## Introduction

A recurring dilemma in clinical practice is whether we should routinely replace central venous catheters (CVCs) to prevent infections or wait and see if one occurs. In haematology patients, peripherally inserted central catheters (PICCs) are frequently used to provide long-term venous access, as they are easier and safer to insert than other types of CVCs [1, 2]. However, like other CVCs, PICCs pose an important, albeit smaller risk for central line-associated bloodstream infection (CLABSI) [3–6], which may lead to hospitalization for intravenous antibiotic therapy [4]. Previous studies have shown that bacterial colonization of intravascular catheters increases with prolonged use, leading to increasing risk of infection with longer indwelling time [5, 7, 8]. This suggests that routine replacement of PICCs may prevent CLABSI.

The Centers for Disease Control and Prevention (CDC) currently advise against routine replacement of CVCs to prevent bloodstream infections, based on results of randomized studies in critically ill patients [2, 9–12]. Unfortunately, results of these studies are not easily translated to the haematology population as CVCs were replaced within several days, whereas PICCs can remain in situ for weeks or even months. In addition, none of the studies investigated PICCs as a type of CVC. Moreover, incidence of CLABSI is higher in patients with aggressive haematological malignancies [13], acute leukaemia in particular [14]. Pre-emptive CVC replacement could thus be beneficial, but studies in this population are lacking.

The value of scheduled replacement of PICCs to reduce risk of infection in patients with haematological malignancies therefore remains an important but unresolved issue. We aimed to assess whether longer indwelling time of PICCs increases the risk of CLABSI in haematology patients. We additionally aimed to describe in detail the microorganisms involved in these infections. The results could aid in determining whether scheduled PICC replacement is useful in reducing CLABSIs in these patients.

## Methods

This cohort study was conducted between June 2013 and June 2015 at the Amsterdam University Medical Centre (location VU University medical centre (VUmc)), a tertiary care centre, and affiliated teaching hospitals in the Netherlands. All PICCs were inserted at the department of haematology in VUmc and were registered in a prospective database designed for quality purposes. Data included previously reported risk factors for CLABSI such as number of catheter days, left or right arm insertion, time needed for insertion, need for repositioning and number of lumens, as well as reasons for removal. Patient data included age, gender, primary haematological diagnosis, use of total parenteral nutrition, highest dose of prednisone in the week before CLABSI and presence of neutropenia. Neutropenia was defined as an absolute neutrophil count or total white blood cell count < 500 cells/mm^3^ on at least two separate days within a seven-day window around a CLABSI event (i.e. 3 days before the event and 3 days thereafter), as proposed by the CDC [15]. Patient data and microbiology data were collected retrospectively from patient records. For logistical reasons, most patients undergoing autologous stem cell transplantation in our centre, are transferred to a regional teaching hospital on the day after stem cell infusion. If patients were transferred while a PICC was in situ, data was obtained from that hospital. PICCs inserted were used for chemotherapy, stem cell transplantations and supportive care. No other types of CVCs than PICCs were used. PICCs inserted for use in non-haematology patients were excluded from the analysis.

### Catheter placement and post-insertion care

All PICCs were inserted by trained nurses. PICC position was assessed through chest radiography; incorrectly positioned catheters were replaced by an intervention radiologist. PowerPICCs (BARD access systems) with an open-ended valve (Bionector®) with heparin lock were used. Antimicrobial lock therapy and antimicrobial catheters were not used during our study. In January 2014, chlorhexidine replaced iodine tincture as disinfectant for PICC placements. Throughout the study period, StatLock® was used for fixation which was routinely replaced every 3 weeks. A chlorhexidine coated dressing (Tegaderm HCG®) was used to seal the insertion site. The dressing and open-ended valve were replaced weekly or earlier if needed. All patients with expected neutropenia of more than 7 days and expected mucositis received antibacterial prophylaxis (ciprofloxacin, amoxicillin or pheneticillin, and oral tobramycin) and antifungal prophylaxis (fluconazole) from start of chemotherapy until recovery of neutropenia. Neutropenic patients with mild or no mucositis received prophylaxis with ciprofloxacin and fluconazole only. High dose steroids may hamper clinical symptoms, leading to delayed onset of antibiotic therapy in patients with bacteraemia [16]. According to hospital protocol, we therefore obtained routine surveillance blood cultures twice weekly in patients treated with high dose corticosteroids for acute GVHD, acute lymphoblastic leukaemia or aplastic anaemia, but not routinely in neutropenic patients.

### Definitions

**Fig. 1:**
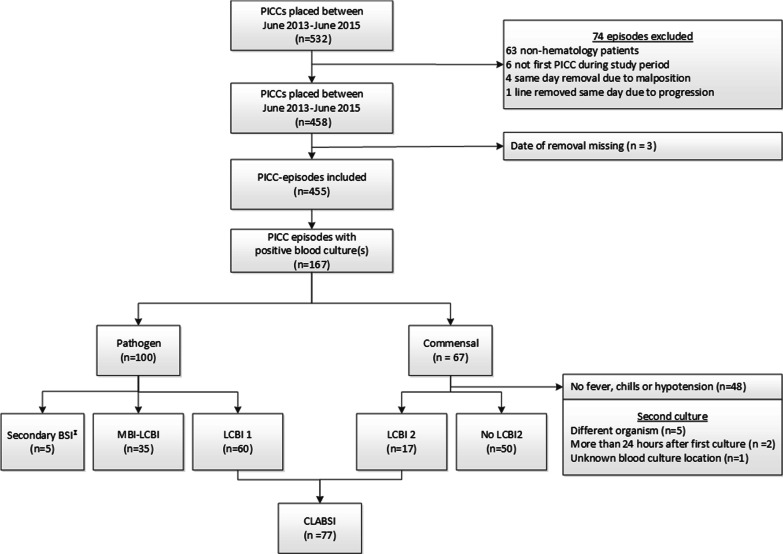
Flow diagram of study selection and description of bloodstream infections (BSI). CLABSI: Central line associated bloodtream infection; LCBI: Labortory confirmed bloodstream infection; MBI: mucosal barrier injury; PICC: peripherally inserted central catheter

CLABSI was defined according to the CDC Device-associated Module BSI ‘Bloodstream Infection Event (Central Line-Associated Bloodstream Infection and Non-central Line-Associated Bloodstream Infection)’ [15]. This module defines CLABSI as a laboratory-confirmed bloodstream infection (LCBI), with either a recognized pathogen (LCBI 1) or a common commensal in the presence of one or more clinical signs or symptoms (temperature > 38.0 °C, chills or systolic blood pressure < 90 mmHg) (LCBI 2, see also Fig. 1). Following CDC recommendations, we excluded secondary bloodstream infections and Mucosal Barrier Injury-LCBI (MBI-LCBI) from CLABSI, as these infections are not related to the central line [17]. How CLABSI was treated, with either antibiotics, PICC removal, or both, was also registered.

### Statistical analysis

We applied descriptive statistics as appropriate. Incidence of CLABSI was calculated per 1000 catheter days. Median time to CLABSI and cumulative incidence of CLABSI were assessed by Kaplan Meier curves and risk factors were investigated using a Cox proportional hazards model. Clinically relevant covariates were retained at the *p* < 0.1 significance level. Using backward elimination, variables with *p*-values < 0.05 were included in the model. To adjust for clustering, sensitivity analyses were performed to assess influence of multiple line episodes per patient on these associations. These analyses were performed with IBM SPSS Statistics (version 22.0). To assess whether the risk of CLABSI increases with longer PICC indwelling time, we calculated incidence rates per 14-day intervals. We used Poisson regression analyses to estimate unadjusted and adjusted incidence rates, using IBM SPSS Statistics (version 26). In the adjusted analyses, we included the clinically relevant covariates that were retained as mentioned above.

### Ethics

The local Medical Ethical Examination Committee determined ethical approval was not required, nor was it needed to obtain consent.

## Results

We evaluated 455 PICC episodes in 351 patients, comprising 19.063 catheter days. Patient and PICC episode characteristics are summarized in Table 1. Diagnoses and treatment characteristics remained constant between 2013 and 2020 (Additional file 1: Table S1). Nearly half of all patients underwent stem cell transplantation (SCT), mostly autologous. A double lumen PICC was placed in the right arm in the majority of patients. Most PICCs were in place for approximately one month. Three episodes were excluded because of missing date of PICC removal.

**Table 1:** Population characteristics

Patient characteristics	
Number of patients	351
Age, *median* (IQR)	58 (50–63)
Gender, *male* (%)	223 (64)

PICC characteristics	
Number of episodes	455
*Hematologic condition, n (%)*	
Multiple myeloma	122 (27)
Acute myeloid leukaemia	118 (26)
Non-Hodgkin lymphoma	103 (23)
Acute lymphoblastic leukaemia	46 (10)
Myelodysplastic syndrome	15 (3)
Hodgkin lymphoma	11 (2)
Aplastic anaemia	10 (2)
Chronic myeloid leukaemia	8 (2)
Chronic myelomonocytic leukaemia	7 (2)
Myeloproliferative neoplasms	6 (1)
Chronic lymphocytic leukaemia	5 (1)
Other^a^	4 (1)
*SCT, n (%)*	220 (48)
Autologous	164 (75)
Allogeneic	56 (25)
*Chemotherapy*	219 (48)
*Supportive care*	16 (4)
*Duration of placement in minutes,* *median* (IQR)^b^	30 (30–45)
Repositioning after placement, n (%)*	126 (28)
*Insertion in right arm, n (%)*	367 (81)
*Number of lumens, n (%)*	
Single lumen	53 (12)
Double lumen	368 (81)
Triple lumen	34 (8)
*PICC line rank, n (%)*	
First	343 (75)
Second	89 (20)
Third or more	23 (5)
*Catheter days, median (range)*	26 (21-57)
*Total parenteral nutrition, n (%)*	52 (11)
Duration in days, *median* (range)	9.5 (6–19)

IQR, interquartile range; SCT, stem cell transplantation

*20 episodes had missing data

^a^Blastic Plasmocytoid Dendritic Cell Neoplasm (n = 2), Systemic sclerosis (n = 1) and Hairy Cell Leukaemia (n = 1)

^b^9 episodes had missing data

^c^*χ*^2^ not significant

### Bloodstream infections

**Fig. 2:**
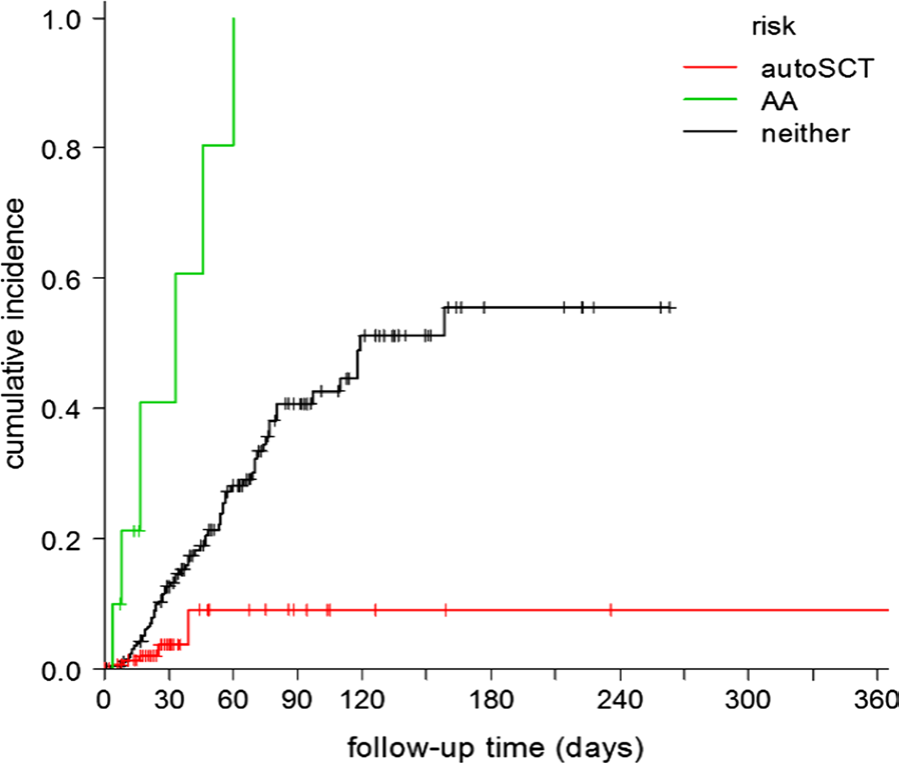
Cumulative incidence of CLABSI. autoSCT: autologous stem cell transplantation (n = 164), AA: aplastic anaemia (n = 10)

CLABSI occurred in 17% (77/455) of PICC episodes in 53 individual patients (out of 351, i.e. 15%), with an incidence of 4.0 per 1000 catheter days (Fig. 1). The median time at risk was 25 days (interquartile range (IQR) (19 to 50) and the median time to CLABSI was 33 days (IQR 18 to 58) (Fig. 2). Forty-eight percent (37/77) of CLABSI cases occurred during neutropenia, none of which were MBI-LCBI, as mentioned previously. Forty percent (30/77) occurred in the outpatient setting; these were all symptomatic. In our cohort, 31% (24/77) of CLABSI events occurred during treatment with steroids. In nine of these (12%), CLABSI was diagnosed by routine surveillance blood cultures in asymptomatic patients. In 33% (10/30) of the outpatient CLABSI events patients had received prophylactic antibiotics. The majority of CLABSI were caused by a recognized pathogen (LCBI1) (Fig. 1). Gram-negative pathogens accounted for 42% (32/77) of bloodstream infections and constituted 53% (48/91) of all cultured pathogens (Table 2). In one third of these episodes the pathogen, predominantly *Pseudomonas* spp., was resistant to ciprofloxacin or tobramycin used as prophylaxis (data not shown). *Staphylococcus epidermidis* and *Enterococcus faecium* were the most prevalent Gram-positive bacteria. If CLABSI occurred, the PICC was removed in 64 of 77 CLABSI events (83%). The median time to PICC-removal after fever onset was 4 days (IQR 2 to14).

**Table 2:** Cultured pathogens in CLABSI

All CLABSI episodes*	91
Pathogen	CLABSI episodes, n (%)
Gram-positive	34 (37)
*Staphylococcus epidermidis*	18 (20)
*Enterococcus faecium*	7 (8)
*Staphylococcus aureus*	3 (3)
*Rothia mucilaginosa*	3 (3)
*Enterococcus faecalis*	3 (3)
*Brevibacterium casei*	2 (2)
Gram-negative	57 (62)
*Stenotrophomonas maltophilia*	7 (8)
*Pseudomonas aeruginosa*	6 (7)
*Acinetobacter spp.*	5 (5)
*Pseudomonas fluorescens*	4 (4)
*Serratia marcescens*	3 (3)
*Escherichia coli*	3 (3)
*Achromobacter spp.*	2 (2)
*Klebsiella pneumoniae*	2 (2)
*Pseudomonas putida*	2 (2)
*Pantoea spp.*	2 (2)
*Brevundimonas diminuta*	2 (2)
*Enterobacter cloacae/asburiae*	2 (2)
Other (all n = 1)**	15 (16)

*In 14 episodes, blood cultures showed 2 different pathogens

**Chrysobacterium indologenes, Citrobacter freundii, Enterococcus casselflavius, Fusarium dimerum, Gemella haemolysans, Micrococcus spp., Mycobacterium chelonae, Neisseria subflava, Ochrobactrum anthropi, Propionibacterium acnes, Pseudomonas oryzihabitans, Rhizobium radiobacter, Sphingomonas paucimobilis, Staphylococcus haemolyticus, Streptococcus pneumoniae

### Risk factors for CLABSI

Univariate analysis revealed that acute lymphoblastic leukaemia, aplastic anaemia, any stem cell transplantation (SCT), but autologous SCT in particular, induction chemotherapy and higher number of lumens had a significant association with CLABSI (Table 3). The multivariate model showed a significant association between aplastic anaemia and occurrence of CLABSI (HR 6.5 [95% CI 2.8–15.2]). Autologous SCT showed an inverse association with the occurrence of CLABSI (HR 0.24 [95% CI 0.10–0.61] (Table 3; Fig. 2)). Sensitivity analyses revealed that the associations remained robust when we excluded multiple line inclusions per patient (n = 85) and subclinical episodes diagnosed by surveillance cultures (n = 9).

**Table 3:** Unadjusted and adjusted analysis of risk factors for CLABSIs in haematology patients

Variables retained in univariate model	Unadjusted HR (95% CI)
AA	8.1 (3.5–19.0)
ALL	1.2 (0.99–3.2)
Autologous SCT	0.22 (0.1–0.6)
Chemotherapy	2.5 (1.4–4.4)
Induction chemotherapy ALL	2.2 (1.0–4.8)
SCT	3.0 (1.6–5.6)
Line rank (2nd vs 1st)	1.6 (0.99–2.7)
Line rank (3rd vs 2nd)	0.15 (0.02–1.1)
NHL	0.60 (0.33–1.1)
Number of lumens (3 vs 2)	2.1 (1.1–4.2)

Variables retained in multivariate model	Adjusted HR (95% CI)
AA	6.5 (2.8–15.2)
Autologous SCT	0.24 (0.10–0.61)

The following variables were not statistically significant in a univariate model: gender, age, acute myeloid leukaemia, Hodgkin lymphoma, multiple myeloma, myelodysplastic syndrome, chronic myeloid leukaemia, chronic myelomonocytic leukaemia, myeloproliferative neoplasm, other malignancies, allogeneic SCT, induction AML, consolidation ALL, induction lymphoma, induction multiple myeloma, side of insertion, duration of insertion procedure, need for repositioning after placement, year of insertion, total parenteral nutrition

AA, aplastic anaemia; ALL, Acute lymphoblastic leukaemia; SCT, stem cell transplantation; NHL, Non-Hodgkin Lymphoma

In the unadjusted analysis, PICCs with a dwell time of 15–28 days, 29–42 days, 43–56 days and > 56 days each had an increased risk of infection compared to PICCs in place for < 15 days (Table 4). After adjusting for aplastic anaemia and stem cell transplantation, there was no significant difference in incidence rates of CLABSIs between dwell times (*p* 0.067).

**Table 4:** Incidence rates and rate ratios of CLABSIs during 14-day intervals after PICC insertion

	Period					
	day 1–14	day 15–28	day 29–42	day 43–56	day > 56	*p*-value for overall analysis
No. of episodes	436	382	180	123	96	
No. of CLABSIs	13	22	10	12	19	
Time at risk in days	5866	4130	2075	1572	4979	
Incidence rate per 1000 catheter days (95% CI)	2.2 (1.3–3.8)	5.3 (3.5–8.1)	4.8 (2.6–9.0)	7.6 (4.3–13.4)	3.8 (2.4–6.0)	
IRR crude (95% CI)	1 (ref.)	2.4 (1.2–4.8)	2.2 (0.95–5.0)	3.4 (1.6–7.5)	1.7 (0.85–3.5)	0.026
IRR adjusted* (95% CI)	1 (ref.)	2.4 (1.2–4.8)	1.8 (0.77–4.1)	2.8 (1.3–6.2)	1.6 (0.80–3.4)	0.067

CI, confidence interval; IRR, incidence rate ratio

*Adjusted for aplastic anaemia and autologous stem cell transplantation

## Discussion

This multicentre cohort study shows that in patients with haematological malignancies, the risk of PICC-associated CLABSI remains constant, regardless of PICC indwelling time. As PICCs in haematology patients usually remain in situ for at least several weeks, this suggests that routine replacement is unlikely to prevent PICC-associated infections and may even be counterproductive, even without taking into account the potential risks and discomfort of scheduled replacement. Our results are in contrast with previous studies that showed non-linear colonization of CVCs, i.e. that the risk of infection increases with longer indwelling time [5, 7, 8] However, CVC colonization in these studies might be different from our population as these studies investigated neonates [5] and intensive care patients or did not include PICCs [7, 8] The indwelling times in these studies (median 14 days, maximum of 15 days in 93% of patients, and median 4–7 days, respectively) were much shorter than in our haemato-oncological population [7, 8]. While external colonization, originating from the skin, is predominant until 10 days after placement, internal colonization, originating from colonization of the hub, increases over time, especially beyond 30 days [18]. Indwelling time might thus alter the type of colonization and possibly the risk of CLABSI. In addition, these studies solely focused on inpatients, whereas our study also included outpatients. In the outpatient setting the PICC is used less intensively (e.g. for drug administration and blood withdrawal), which might decrease the risk of bacterial translocation to the bloodstream.

CLABSI incidence in our study was 4.0 per 1000 catheter days, which is in line with previous studies in haematology patients with PICCs (incidence rates of 0.17 to 6.61/1000 catheter days) [6, 14, 19, 20]. In our cohort, 42% of CLABSI were due to Gram-negative pathogens, even though all these patients received prophylactic antibiotics. This is in contrast with earlier epidemiologic studies in haematology patients reporting Gram-positive pathogens as the most common cause for bloodstream infection (55%, mostly coagulase negative staphylococci (CNS). Although a more recent study suggested an increase in Gram-negative bacteria [21], this shift could also be due to the CDC-definition of CLABSI which is influenced by the strict definition of secondary bloodstream infection from the gastrointestinal tract (MBI-LCBI). This definition leads to the less frequent diagnosis of secondary bloodstream infection due to mucosal barrier disruption, because this diagnosis is only allowed when grade 3 or 4 intestinal graft versus host disease is present or if neutropenia was present during the three days before and after the date of bacteraemia. Because of this definition, some secondary BSI, such as MBI-LCBI, could be diagnosed as CLABSI. Hence, the CLABSI incidence in our study may be an overestimation as some CLABSI events could actually have been caused by mucosal barrier injury [22].

In our cohort there was only one bloodstream infection with *Candida kruseï*, constituting an incidence of 0.22%: 1 of 455 PICC episodes during the study period. This is low compared to previously reported incidences of 0.4% to 1.4% [23, 24]. Only a minority of patients in our cohort had acute myeloid leukaemia (AML) or myelodysplastic syndrome (MDS) (known risk factors for candidemia due to the severity of mucositis and the long duration of neutropenia), which could explain our low incidence. In addition, almost all patients in our cohort received prophylaxis with fluconazole, which may have protected them from candidemia.

One third of Gram-negative blood culture isolates showed resistance against standard prophylaxis, which might have led to breakthrough Gram-negative bacteraemia.

We identified aplastic anaemia (AA) as an important risk factor for CLABSI. Patients with AA typically have prolonged neutropenia and receive immunosuppressive therapy that includes high doses of steroids, which could make these patients more prone to CLABSI. As mentioned before, surveillance blood cultures were obtained in these patients, which may have led to higher detection of bacteraemia. However, this is less likely with the crude CLABSI definitions, in which positive blood cultures with common commensal bacteria (such as CNS) are only considered CLABSI if clinical symptoms (fever, chills, and/or hypotension) are present. Autologous stem cell transplant recipients on the other hand appeared to have a lower risk of CLABSI, which may be due to the short duration of neutropenia and consequently shorter PICC indwelling time.

Although total parenteral nutrition (TPN) has previously been described as a risk factor for CLABSI in different subgroups such as trauma and ICU patients, and patients with gastro-intestinal malignancies, we did not find this association in our cohort. In patient with haematological malignancies, TPN is used to overcome alimentary problems due to nausea and mucositis. Compared to non-haematology patients, TPN is used for a relatively short period of time (a median duration of 9.5 days in our cohort) (Table 1), which may explain these differences. Unfortunately, most studies report TPN as a binary variable and do not report median duration of TPN.

Our study has strengths and limitations. To our knowledge, this is the first study to investigate whether the risk of PICC-associated CLABSI increases with longer indwelling time in a high-risk population of haematology patients. Our cohort represents a diverse group with respect to age, gender, and primary haematological diagnosis, and included a large cohort over several years in multiple hospitals. Diagnoses, treatment characteristics and infection prevention policies remained constant between 2013 and 2020. Therefore our findings can be applied to the current situation. Our study is subject to the general limitations of an observational design, which means that information bias may have been introduced: although most of our database was kept prospectively, post-insertion data from other hospitals was collected retrospectively, which may have led to misclassification. Antibiotic prophylaxis was used in all our patients which is not standard of care in all haematology centres and could impair generalisability. Furthermore, although antibiotic prophylaxis may be a confounder in the association between PICC indwelling time and CLABSI, we did not include this as a variable. This is due to the fact that it is frequently discontinued en later resumed during the course of treatment and it is not known at which point in time antibiotic prophylaxis may or may not have prevented CLABSI, especially for patients in whom no CLABSI occurred. This means there is no reliable way to adjust for antibiotic prophylaxis. In retrospective research, one must always consider the possibility of a small true difference that is not detected due to sample size. The incidence of CLABSI in our cohort and the total number of catheter days included does make this less likely.

We assessed the occurrence of CLABSI based on CDC-definitions, which is a rigorous method and therefore adds to generalisability. However, as the CDC guideline was mainly developed for surveillance purposes, definitions of CLABSI are rather stringent, which means that clinically suspected catheter-related infections without positive blood cultures are not reported. As suspected CLABSI is an important driver of antibiotic use in clinical practice, this method may have underestimated clinically relevant incidence of CLABSI.

## Conclusions

Our study indicates that in patients with haematological malignancies, prolonged indwelling time of PICCs does not lead to an increased risk of CLABSI. Routine replacement may therefore not be necessary in this population.

## Supplementary Information

**Additional file 1: Table S1**. Annual figures of PICCs, diagnoses and SCT from 2013 to 2020.

## Abbreviations

PICC: Peripherally inserted central catheters
CLABSI: Central line associated bloodstream infections
AA: Aplastic anaemia
SCT: Stem cell transplantation
CVCs: Central venous catheters
CDC: Centers for Disease Control and Prevention
VUmc: VU University medical centre
LCBI: Laboratory-confirmed bloodstream infection
MBI-LCBI: Mucosal barrier injury-LCBI
CNS: Coagulase negative staphylococci
AML: Acute myeloid leukaemia
MDS: Myelodysplastic syndrome
TPN: Total parenteral nutrition
