# Supplementary material for: Indwelling time of peripherally inserted central catheters and incidence of bloodstream infections in haematology patients: a cohort study
Source: Antimicrob Resist Infect Control. 2022 Feb 17;11:37. doi: 10.1186/s13756-022-01069-z (PMC8851849; doi:10.1186/s13756-022-01069-z)
Supplement: Supplementary file 1 — Additional file 1: Table S1. Annual figures of PICCs, diagnoses and SCT from 2013 to 2020. [file 13756_2022_1069_MOESM1_ESM.docx]

**SUPPLEMENTAL TABLE S1** Annual figures of PICCs, diagnoses and SCT from 2013-2020

| Year | No. PICCs placed | AML/MDS (% of PICCs) | MM (% of PICCs) | Lymphoma (% of PICCs) | Autologous SCT | Allogeneic SCT |
| --- | --- | --- | --- | --- | --- | --- |
| 2013-2015 | 744 (248/year) | 209 (28) | 169 (23) | 134 (18) | 295 (98/year) | 150 (50/year) |
| 2016-2020 | 1661 (332/year) | 575 (35) | 379 (23) | 217 (13) | 689 (137/year) | 259 (52/year) |

AML: acute myeloid leukaemia; SCT: stem cell transplantation MDS: myelodysplastic syndrome; PICC: Peripheral inserted central catheter;
